# Genital Microbiota of Women From Six Ethnic Groups With and Without Human Papillomavirus Infection in Shangri-La, China

**DOI:** 10.3389/fcimb.2022.935068

**Published:** 2022-07-08

**Authors:** Chen-Jian Liu, Wen-Yu Xiao, Jun-Feng Fang, Yong-Hong Dong, Ke-Fan Ye, Meng-Ping He, Yan-Song Wang, Xiao Li, Zhi-Min Zhao, Tao Yuan, Ting Zhao, Chun-Yan He, Shu-Ming Zhang, En Yang, Xiao-Mei Wu, Xiao-Ran Li

**Affiliations:** ^1^ Faculty of Life Science and Technology, Kunming University of Science and Technology, Kunming, China; ^2^ Gynaecology Department, The First People’s Hospital of Yunnan Province, Kunming, China; ^3^ Gynaecology Department, The Affiliated Hospital of Kunming University of Science and Technology, Kunming, China; ^4^ Obstetrics and Gynecology Department, Diqing Tibetan Autonomous Prefectural People’s Hospital, DiQing, China

**Keywords:** genital microbiota, vagina, cervix, gut microbiota, HPV

## Abstract

**Background:**

A diversity of microorganisms is associated with human health and exists in a state of dynamic equilibrium. This diversity has direct implications for the assessment of susceptibility to infectious diseases, especially human papillomavirus (HPV) infection.

**Methods:**

Here, we investigated the relationships between HPV infection and vaginal, cervical, and gut microbiota composition and assessed the levels of genital immune mediators. We selected a multiethnic area in Yunnan Province, China, to collect samples from healthy women of childbearing age. A total of 82 healthy women of childbearing age were included in this study. Vaginal, cervical, and rectal swabs were collected to analyze the microbial community, and cytokines were analyzed in some samples.

**Findings:**

Different proportions and types of HPV infection were detected in cervical (44%), vaginal (18%), and rectal (18%) swabs. HPV detected in cervical swabs was generally a high-risk type, while low-risk HPV types were primarily detected in vaginal and rectal swabs. There were some differences in this proportion as well as in the microbial community composition among different ethnic groups. Rectal samples exhibited the highest diversity index, while vaginal samples displayed the lowest diversity index. *Lactobacillus* dominated most of the vaginal samples, was decreased in HPV-positive samples, and differed among different ethnic groups. However, the sequence proportion of *Lactobacillus* in the cervix exhibited the opposite trend in those affected by HPV infection. The dynamic balance between the potential pathogens *Gardnerella* and *Lactobacillus* determines the health of the female genital system.

**Interpretation:**

This study constitutes the first step toward personalized medicine for women’s reproductive health, wherein differences between the genital microbiomes of individuals would be considered in risk assessment and for subsequent disease diagnosis and treatment.

## 1 Introduction

Microbial communities in the human vagina play an integral role in maintaining women’s genital health (Nunn et al., 2020), comprising a diverse array of beneficial microbes and opportunistic pathogens that inhabit the vaginal milieu (Smith and Ravel, 2017; Castanheira et al., 2021). The composition of the vaginal microbiome is influenced by various factors, such as ethnicity, hormonal alterations, sexual activity, and hygiene habits, as well as lactation, diabetes mellitus, stress, and dietary factors (Amabebe and Anumba, 2018; McKee et al., 2019). Some studies have shown that the vaginal microbiome differs among women of different ethnicities (Zhou et al., 2007; Norenhag et al., 2020). Such diversity may be related to genetic differences among races, including a few mitochondrial DNA haplotypes. This reveals the importance of genetic factors in determining the microbiome of individuals, making them more or less prone to various diseases (Laniewski et al., 2018). The vaginal ecosystem possesses its own characteristics, but it is in correlation with other border ecosystems such as the urinary, intestinal, and cutaneous systems (Palma and Bertini, 2019).

High-throughput studies addressing the diversity and abundance of the vaginal microbiota in the female genital tract have shown that several factors, including hormonal levels, hygiene habits, and sexually transmitted diseases, may disrupt the natural balance, favoring the outgrowth of some groups of bacteria, which in turn may favor some pathological states (Laniewski et al., 2020; Castanheira et al., 2021). Vaginal dysbiosis reflects the disruption of the microbial community in the vagina and has been frequently associated with several gynecological diseases. Moreover, a possible mechanism for vaginal dysbiosis involves an increase in the production of proinflammatory cytokines and chemokines associated with an increase in pathogenic microbial diversity, which contributes to the additional recruitment of immune cells and amplification of the inflammatory response (Torcia, 2019). The microbiome of a healthy female genital tract is characterized by the presence of one or few varieties of lactobacilli. Specific bacteria, such as *Gardnerella*, and increased microbiological diversity may represent biomarkers of cervical changes to identify women with a high risk of developing persistent human papillomavirus (HPV) infection or even cancer (Usyk et al., 2020).

HPVs are non-enveloped small DNA viruses that widely exist in nature and encompass a large family of viruses, containing both benign and highly carcinogenic members (Van Doorslaer et al., 2018). Currently, more than 170 types of HPV have been discovered, and >40 of these are associated with female reproductive tract infections and cervical lesions (Oliver et al., 2017). HPV subtypes can be broadly divided into high- and low-risk groups according to their differences in pathogenicity. Low-risk types, such as HPV6, HPV11, and HPV30, can cause benign lesions, such as lower-grade squamous intraepithelial lesions (LSIL) and genital warts, whereas high-risk types, such as HPV16, HPV18, and HPV58, primarily cause higher-grade squamous intraepithelial lesions (HSIL) or lead to cervical cancer (CC) (Yang et al., 2020). The contribution of the microenvironment of the vaginal and cervical microbiota to the occurrence and development of cervical precancerous lesions has gained increased interest in recent years.

CC represents the fourth most frequent malignant neoplasm among women worldwide and is a serious public health problem. In 2018, approximately 570,000 new cases and 311,000 deaths occurred, most of which were in developing countries (Cohen et al., 2019; Zhou et al., 2019). Persistent infection with HPV subtypes of high-oncogenic-risk HPV (hrHPV) is the primary factor influencing the development of CC and has been observed in 99.7% of CC samples (Graham, 2017). However, HPV is necessary but not sufficient to cause CC. In most infected women, the immune response is able to control the infection and prevent high-grade lesions and tumors (Kovachev, 2020). Among the cofactors in CC development, the vaginal microbiota may play an important role (Kwasniewski et al., 2018).

Shangri-La is a multiethnic (a total of 34 ethnic groups) inhabited area in Southwest China. The first six dominant ethnic groups are Zang, Naxi, Han, Lisu, Yi, and bai ethnic groups. Although people in Shangri-La experience beautiful scenery and quiet lives, compared to China’s metropolis, the levels of local education, science, and technology are relatively backward. One phenomenon worthy of attention is that according to incomplete statistics of local hospitals, the incidence rate of cervical cancer among local women of childbearing age is very low, which is a problem worthy of attention, and the real reason is worth investigating. First, to gain a deeper understanding of the genital tract health of fertile women in the region, we held a health screening in the summer of 2020, aiming to gain a deeper understanding of the genital tract health of fertile women in Shangri-La based on the study of vaginal and cervical microorganisms (bacteria, archaea, and HPV). Then, we performed an in-depth assessment of the composition and ecology of the vaginal microbial ecosystem in asymptomatic women. Gut microbial communities were used to assess the effects of the border ecosystems on reproductive system microbes. The data obtained are an essential prerequisite for understanding the role and function of the vaginal microbiota in reducing the risk of acquiring disease and identifying the factors that determine disease susceptibility. At the same time, due to the individual differences of the human microbiome, this study would also provide the first step for personalized medical treatment of female reproductive health.

## 2 Materials and methods

### 2.1 Sample Collection and Study Design

This study was approved by the medical ethics committee of the First People’s Hospital of Yunnan Province (2018GJ037). All subjects provided written informed consent, and there was no financial compensation. A total of 82 healthy women of childbearing age were included in this study. All samples were collected at Diqing People’s Hospital, Yunnan Province, China. All subjects had to meet the following requirements: 1) no use of antibiotics within 1 week, 2) no vaginal medication or flushing within 1 week, 3) no obvious vaginal symptoms, and 4) normal sexual life.

Eleven swab samples were collected from each subject (three repetitions of vaginal, cervical, and rectal swabs and duplicate blood samples, of which 39 patients did not provide blood samples). The participants completed relevant investigation questionnaires, including age, education level, occupation, economic status, health habits, sexual activities, gynecological history, and understanding of HPV. Then, in the women’s examination room, the medical staff collected swabs from the cervix, vagina, and rectum. Venous blood collection was performed after swab collection. Blood samples were centrifuged in a 4°C cryogenic centrifuge at 8,000 rpm for 10 min to separate serum. Five swabs were also exposed to air for the same time as the negative controls. All samples were stored in a -80°C freezer and then transported back to the laboratory with a refrigerant for further treatment.

### 2.2 Whole-Genomic DNA Extraction From Swabs and PCR Amplification and Sequencing

In the laboratory, all samples were renumbered, including negative controls, to eliminate human influence. Total DNA from swab samples was isolated using the QIAamp PowerFecal DNA Kit (Qiagen, Hilden, Germany) for PCR amplification and HPV genotyping. The archaeal and bacterial 16S rRNA genes were amplified using the primer set 515F (Reysenbach et al., 1992) and 909R (Brunk and Eis, 1998). A 10-ng aliquot of each sample was used for PCR with Rapid *Taq* Master Mix (Vazyme, Nanjing, China), and the annealing temperature was 51°C. Amplicons were cleaned using the UltraClean PCR Cleanup Kit (MOBIO, Carlsbad, CA, USA), and an equivalent amount of PCR product was mixed for sequencing using the Illumina MiSeq™ system (Illumina, San Diego, CA, USA).

### 2.3 Bioinformatics Analysis

All sequences were demultiplexed using the barcodes of each sample. The primer sequences were cut down by employing pairwise sequence alignment, and sequences were gathered to correct for sequencing errors. Sequence processing was performed by combining features of Mothur v1.44.1 (Schloss et al., 2009) according to MiSeq SOP. The SSU rRNA database sequences and taxonomic information from SILVA (v138) (Pruesse et al., 2007) were downloaded directly from the Mothur website. Chimera checking was performed after the sequences were aligned using the UCHIME algorithm (Edgar et al., 2011). Similar sequences were clustered into operational taxonomic units (OTUs) with a minimum identity of 97% or 100% according to different analyses. The distance matrix was analyzed using the thetaYC and JClass (Schloss et al., 2009) methods, and the larger principal components were selected for principal component analysis (PCA). Groups are clustered using the unweighted pair group method with arithmetic means (UPGMA) algorithm using the distance between communities as calculated using any of the calculators describing the similarity in community membership. Population levels between different groups of samples were analyzed using Metastats and Lefse, and the effect relevance was predicted using linear discriminant analysis (LDA) (Segata et al., 2011). The community types were defined based on Dirichlet multinomial mixtures, as described by Holmes et al., and this approach was used because it allows for clustering from unevenly sampled populations (Holmes et al., 2012). A non-parametric analog analysis of molecular variance (AMOVA) was used to test the hypothesis that genetic diversity within two populations is not significantly different from that which would result from pooling the two populations. Phylogenetic trees of sequences from each OTU as well as the genera *Lactobacillus* and *Gardnerella* were constructed using MEGA version 6.0 with the neighbor joining method using 1,000 bootstraps.

### 2.4 Statistical Analysis of Demographic Data

SigmaPlot v11.0 was used for data analysis. Age was analyzed using analysis of variance (ANOVA), and the other items were analyzed using the chi-square test or Fisher’s exact probability test. The chi-square test was used to test categorical variables. To identify a significant difference in alpha diversity, the Kruskal–Wallis rank-sum test was used to evaluate the differences in diversity among the three groups, followed by Dunn’s test of multiple comparisons. To analyze genus differences, the Mann–Whitney test and Wilcoxon signed-rank test were used for the comparative analysis within and between the vaginal and cervical microbiota, respectively. All samples were clustered to low, medium, and high levels to determine the correlation coefficients between individual cytokines according to cytokine levels (logarithm of 10) by average linkage cluster. p < 0.05 was considered statistically significant.

### 2.5 Nucleotide Sequence Availability

The PCR product sequencing data of the article entitled “Genital microbiota of reproductive-age women from six ethnic groups with and without human papillomavirus (HPV) infection in Shangri-La, China” had been uploaded to The National Omics Data Encyclopedia at https://www.biosino.org/node/search, accession number: OEP002667.

### 2.6 Quantification of Bacteria and Lactobacillus

The copy numbers of the total 16S rRNA gene in bacteria (Bahl et al., 2012) and the 16S rRNA gene of the genus *Lactobacillus* (Byun et al., 2004) were determined in each sample using qPCR according to the instructions. The specificity of the amplification was determined by melting curve analysis and gel electrophoresis. Cycle thresholds were determined *via* comparison with standard curves constructed using *Escherichia coli* strain B (Sigma, St. Louis, MO, USA) for the 16S rRNA gene of total bacteria and *L. plantarum* AY01 (Li et al., 2013) for the genus *Lactobacillus*. The relative copy number of three replicates of each sample was evaluated for each target organism.

### 2.7 HPV Genotyping

Type-specific HPV viral load measurement and genotyping were simultaneously conducted using the HPV GenoArray Test Kit (Hybribio Ltd., China) to detect 37 different types of HPV in patient specimens, comprising 18 high-risk HPV types (HPV 16, 18, 31, 33, 35, 39, 45, 51, 52, 53, 56, 58, 59, 66, 68, 26, 73, and 82) and 19 low-risk HPV types (HPV 6, 11, 34, 40, 42, 43, 44, 54, 55, 57, 61, 67, 69, 70, 71, 72, 81, 83, and 84).

### 2.8 Measurement of Cytokine Concentrations

The collected blood was centrifuged at 8,000 rpm in a cryogenic centrifuge for 10 min, and the supernatant serum was stored at −20°C until cytokine measurement. The concentrations of nine cytokines, interleukin (IL2, IL4, IL6, IL8, IL10, IL12P70, and IL23), INF-γ, and TNF-α, were measured using ELISA kits (Shanghai Enzyme-Linked Biotechnology Co., Ltd., China).

## 3 Results

### 3.1. Demographics

A total of 100 volunteers were willing to participate in this study. After removing the samples that do not belong to six ethnic groups, a total of 82 subjects were used in this study. To characterize the cervical and vaginal microbiota in women living in Shangri-La, we obtained vaginal, cervical, and rectal swab samples from 82 subjects from six ethnic groups. Subjects in each group were age matched (**Table 1**, p = 0.906). The living habits of women from the six ethnic groups were roughly similar. The average age of all women was 39.7 ± 7.1. A total of 14.6% of women had vaginal symptoms (such as abnormal leucorrhea and pruritus), while Yi, Bai, and Lisu women did not have vaginal symptoms. Only Yi women did not have uterine or cervical symptoms, and the percentage among all samples with uterine or cervical symptoms was 12.2%. Women with a high level of education were mostly engaged in government work. Only two women were smokers. There were some differences in the number of pregnancies among women of different ethnic groups. Yi women had the highest pregnancy numbers, while Naxi women had the lowest number. The higher the level of education and income, the fewer pregnancies were observed. Five women experienced episiotomy. A total of 37.8% of women experienced abortion, and 58% of women experienced dystocia.

**Table 1:** Demographics of participants.

	Zang (N = 42)	Naxi (N = 13)	Yi (N = 7)	Bai (N = 4)	Lisu (N = 6)	Han (N = 10)	p
Mean age (year)	39.5 ± 7.2	38.9 ± 7.3	41.4 ± 4.5	37.8 ± 6.6	39.8 ± 9.7	41.6 ± 7.2	0.906
BMI	24.2 ± 3.1	23.4 ± 3.1	23.1 ± 2.1	23.7 ± 1.3	22.1 ± 1.4	23.6 ± 3.0	0.365
Vaginal symptoms or not	5 (12)^1^	2 (15)	0	0	0	5 (50)	0.108
Uterine or cervical symptoms or not	3 (7)	2 (15)	0	1 (25)	2 (33)	2 (20)	0.011
Educational level							0.970
Non-educated	1 (2)	0	3 (43)	0	2 (33)	0	–
Primary school	16 (38)	1 (8)	4 (57)	0	0	2 (20)	–
Middle school	15 (36)	3 (23)	0	2 (50)	3 (50)	6 (60)	–
Bachelor	9 (21)	8 (62)	0	2 (50)	1 (17)	2 (20)	–
≥ Master	1 (2)	0	0	0	0	0	–
Monthly income (¥)							0.993
<1,000	1 (2)	0	0	0	0	1 (10)	–
1,000–3999	19 (45)	2 (15)	5 (71)	0	2 (33)	4 (40)	–
>4,000	22 (52)	11 (85)	2 (29)	4 (100)	4 (67)	5 (50)	–
Occupation							0.993
Medical service	2 (5)	0	0	0	0	0	–
Farmer or housewife	19 (45)	0	1 (14)	0	0	4 (40)	–
Civil servant	12 (29)	10 (77)	0	3 (75)	3 (50)	5 (50)	–
Others	9 (21)	3 (23)	6 (86)	1 (25)	3 (50)	1 (10)	–
Smoking or not							0.910
Yes	1 (2)	0	1 (14)	0	0	0	–
No	41(98)	13 (100)	6 (86)	4 (100)	6 (100)	10 (100)	–
Number of pregnancies	2.2 ± 1.0	1.5 ± 1.1	3.3 ± 1.8	1.8 ± 0.5	2.2 ± 1.0	2.4 ± 1.6	0.105
Episiotomy							0.100
Yes	2 (5)	2 (15)	0	1 (25)	0	0	
No	40 (95)	11 (85)	7 (100)	3 (75)	6 (100)	10 (100)	
Number of abortion							0.100
No	28 (67)	8 (62)	5 (71)	2 (50)	2 (33)	6 (60)	
≥ 1 times	14 (33)	5 (38)	2 (29)	2 (50)	4 (67)	4 (40)	
Experience of dystocia							0.100
Yes	33 (79)	6 (46)	6 (86)	3 (75)	6 (100)	4 (40)	
No	9 (21)	7 (54)	1 (14)	1 (25)	0	6 (60)	

^1^Percentages.

HPV 16/18 and other high-risk subtypes were specifically separated, and some of the enrolled subjects were infected with multiple subtypes (**Table 2**). The prevalence of HPV 16 and 18 was 43% and 1%, respectively, in which some subjects were coinfected with both. Only two women were infected with multiple HPV subtypes. Compared to the high HPV detection rate of cervical samples (44%), the detection rate of the vaginal (18%) and rectal (18%) samples was lower, but the types of HPV were more common than in cervical samples. In cervical samples, there was no significant difference in HPV infection rate among different ethnic groups (p > 0.05), while the HPV infection rates from vaginal and rectal swabs were significantly different among the six ethnic groups. In general, HPV detected in cervical swabs was generally a high-risk type, while low-risk HPV was primarily detected in vaginal and rectal swabs.

**Table 2:** Type-specific HPV prevalence by cytology triage test result.

Nationalities		Zang (N = 42)			Naxi (N = 13)			Yi (N = 7)			Bai (N = 4)			Lisu (N = 6)			Han (N = 10)			Total (n = 82)		
Sampling site		C ^1^	V ^2^	R ^3^	C	V	R	C	V	R	C	V	R	C	V	R	C	V	R	C	V	R
	Type (s)	n(%)	n(%)	n(%)	n(%)	n(%)	n(%)	n(%)	n(%)	n(%)	n(%)	n(%)	n(%)	n(%)	n(%)	n(%)	n(%)	n(%)	n(%)	%	%	%
High-risk	HPV 16	17 (40)	3 (7)	0	8 (62)	1 (8)	1 (8)	2 (29)	0	0	2 (50)	0	0	2 (33)	0	0	4 (40)	0	0	43	5	1
	HPV 18	1 (2)	0	1 (2)	0	0	0	0	0	0	0	0	0	0	0	0	0	0	0	1	0	1
	16 and 18	17 (40)	3 (7)	1 (2)	8 (62)	1 (8)	1 (8)	2 (29)	0	0	2 (50)	0	0	2 (33)	0	0	4 (40)	0	0	43	5	2
	HPV 39	0	0	1 (2)	0	0	0	0	0	0	0	0	0	0	0	0	0	0	0	0	0	2
	HPV 51	1 (2)	1 (2)	1 (2)	1 (8)	1 (8)	1 (8)	0	0	0	0	0	0	0	0	0	0	0	0	2	2	2
	HPV 52	0	1 (2)	1 (2)	0	0	0	0	0	0	0	0	0	0	0	0	0	0	0	0	2	2
	HPV 53	0	0	1 (2)	0	0	1 (8)	0	0	0	0	0	0	0	0	0	0	1 (10)	0	0	1	2
	HPV 56	0	0	0	0	1 (8)	0	0	0	0	0	0	0	0	0	0	0	0	0	0	1	0
	HPV 66	0	0	1(2)	0	0	0	0	0	0	0	0	0	0	0	0	0	0	0	0	0	1
	HPV 68	0	1 (2)	0	0	0	1 (8)	1 (14)	1 (14)	2 (29)	0	0	0	0	0	0	0	0	0	1	2	4
Low-risk	HPV 34	0	1 (2)	1 (2)	0	0	0	0	0	0	0	0	0	0	0	0	0	0	0	0	2	2
	HPV 40	0	0	2 (5)	0	0	0	0	0	1 (14)	0	0	1 (25)	0	0	0	0	0	0	0	0	4
	HPV 44	0	1 (2)	1 (2)	0	0	0	0	0	0	0	0	0	0	0	0	0	0	0	0	2	2
	HPV 54	0	2 (5)	1 (2)	0	0	0	0	1 (14)	1 (14)	0	0	0	0	0	0	0	0	0	0	4	2
	HPV 61	0	1 (2)	0	0	1 (8)	0	0	0	1 (14)	0	0	0	0	0	0	0	0	0	0	2	1
	HPV 67	1 (2)	0	0	0	0	0	0	0	0	0	0	0	0	0	0	0	0	1 (10)	1	0	1
	HPV 69	0	0	1 (2)	0	0	0	0	0	0	0	0	0	0	0	0	0	0	0	0	0	1
	HPV 84	0	0	1 (2)	0	0	0	0	0	0	0	0	0	0	0	0	0	0	0	0	0	1
	No. of women infected ^4^	17 (40)	9 (23)	9 (23)	8 (62)	3 (23)	2 (15)	3 (43)	2 (29)	2 (29)	2 (50)	0	1 (25)	2 (33)	0	0	4 (40)	1 (10)	1 (10)	43	18 ***	18 *

1 Cervical swabs.

2 Vaginal swabs.

3 Rectal swabs.

4 Numbers of people infected.

### 3.2 Differences in Genital Microbial Community Structure Among Different Ethnic Groups in Shangri-La

#### 3.2.1 Alpha diversity index

**Figure 1:**
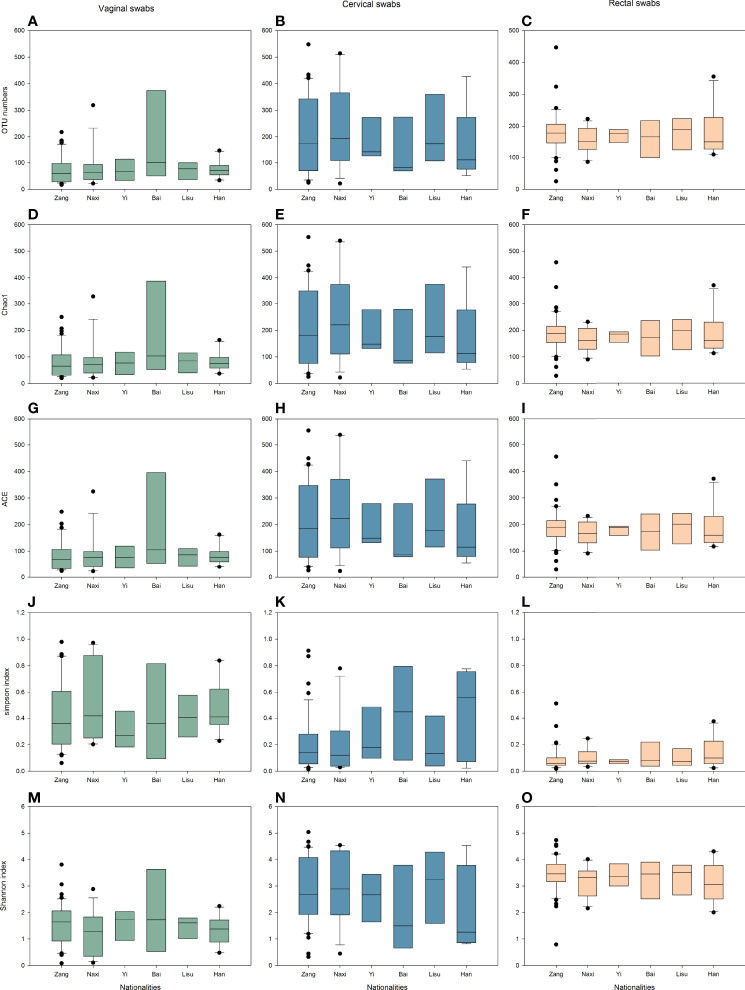
Alpha diversity indices of vaginal (green), cervical (blue), and rectal (yellow) microbiota. **(A–C)** OTU numbers. **(D–F)** Chao1 indices. **(G–I)** ACE indices. **(J–L)** Simpson indices. **(M–O)** Shannon indices.

To estimate the differences in microbial diversity among the six ethnic groups, alpha diversity based on the number of operational taxonomic units (OTUs) and the Chao1 ACE, Simpson and Shannon indices were determined and are shown in **Figure 1**. We observed that biodiversity was highest in the rectal swabs and was higher in cervical swabs than in vaginal swabs. Among different individuals, the difference in alpha diversities of cervical swabs was the largest, followed by vaginal swabs, while the individual difference in rectal swab samples was very small. The lower Simpson index also confirmed similar results in evenness and richness. The diversity index was similar among all samples from the six ethnic groups.

#### 3.2.2 Microbial Community Composition at the Phylum Level

**Figure 2:**
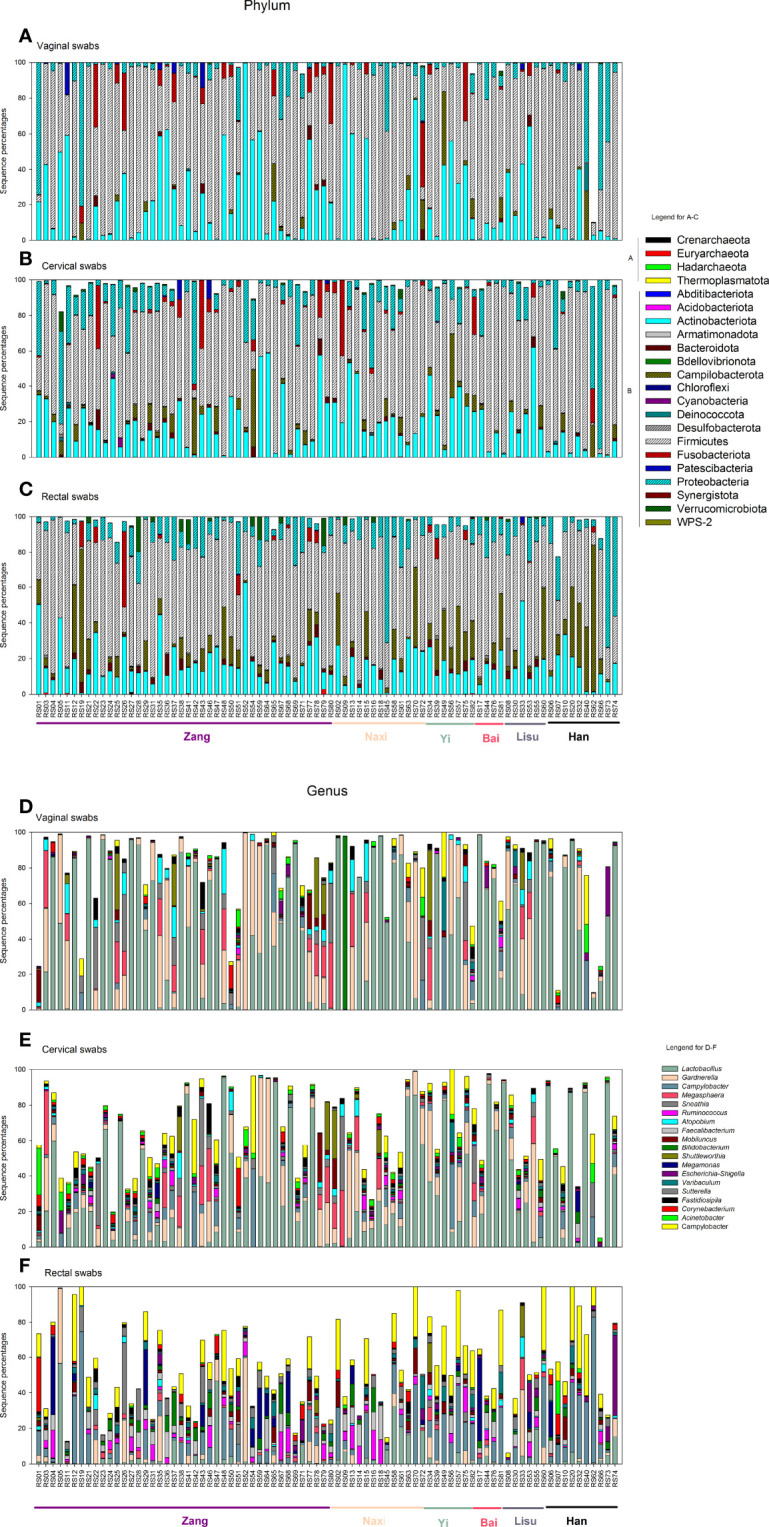
Vaginal, cervical, and rectal microbiota distribution. **(A–C)** Vaginal, cervical, and rectal microbiota distribution at the phylum level. **(D–F)** Vaginal, cervical, and rectal microbiota distribution at the genus level. **(A, B)** in panels **(A–C)** indicate the phyla belonging to Archaea and Bacteria. Different ethnic groups are marked in the bottom of the columns with different colors (Zang: purple; Naxi: skin; Yi: mint green; Bai: rose red; Lisu: dark gray; Han: black).

A total of 22 phyla and 890 genera were detected in this study, including archaea and Bacteria (**Figure 2**). A total of 0.04% of sequences belonged to the Archaea domain. We detected a small number of Archaea sequences in 18 rectal swabs. In vaginal swabs, sequences belonging to archaea were detected in 5/82 samples, while the percentage was 15/82 in cervical swabs.

The Archaeal genus *Methanobrevibacter* was detected in one subject from all three sampling sites. In Bacteria, *Lactobacillus* was still the dominant genus in vaginal (44%) and cervical (32%) samples (**Figures 2D** **, E**), while the *Campylobacter* genus was the most abundant in rectal swabs (**Figure 2F**). The Bai and Lisu women exhibited higher *Lactobacillus* abundance, while Yi displayed the lowest abundance in the vaginal samples. *Gardnerella* was the second most abundant genus in vaginal (15%) and cervical (10%) samples. In vaginal swabs, Bai and Han women had lower percentages of the genus *Gardnerella*, while Lisu had the highest percentages.

#### 3.2.3 OTU-Based Analysis

**Figure 3:**
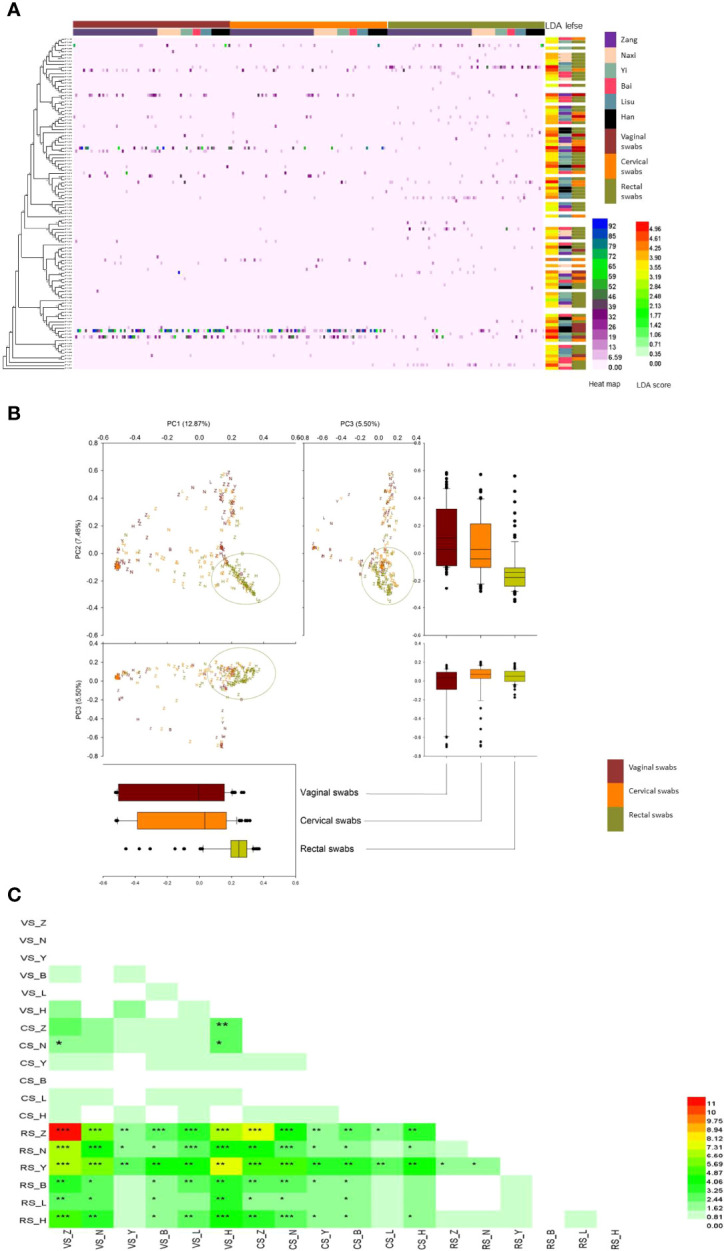
Comparison of the microbiomes of vaginal, cervical, and rectal swab samples among six ethnic groups. **(A)** Heatmap and phylogenetic analysis of the 107 most abundant OTUs (sequence percentages > 0.5%) at a 0.03 cutoff in all samples. OTUs that were significantly elevated are shown in different colors in the right column of the heatmap calculated using LDA scores. OTUs that were significantly elevated as calculated by LEfSe analysis are shown in different colors according to the six ethnic groups (the right column next to the LDA scores) and vaginal, cervical, and rectal swabs (the column on the far right). Color legend: vaginal swabs, dark brown; cervical swabs, orange; rectal swabs, mustard green; Zang, purple; Naxi, skin; Yi, mint green; Bai, rose red; Lisu, dark gray; Han, black. **(B)** PCA plot based on relative taxon abundance. Samples are marked by the group type. The boxplots represented the distribution of PC values corresponding to the coordinate axis. Each ethnic group is represented by its initials. Color legend: vaginal swabs, dark brown; cervical swabs, orange; rectal swabs. **(C)** AMOVA distance matrix between each pair of samples. *p < 0.05, **p < 0.01, ***p < 0.001. VS, vaginal swabs; CS, cervical swabs; RS, rectal swabs. Each ethnic group is represented by its initials.

After quality filtering, a total of 7,461 OTUs based on 97% sequence similarity were achieved from 243 swab samples. Detailed information and specific distributions for the top 107 OTUs (sequence percentages > 0.5%) in all samples are shown in **Figure 3A**. The distribution of major OTUs in the genital tract was significantly different from that in the gut. Among the top 10 OTUs named by OTU abundance, the OTUs primarily distributed in the vaginal and cervical swab samples were OTU0001 (*Lactobacillus iners*), OTU0002 (*L. acidophilus*), OTU0003 (*Gardnerella leopoldii*), OTU0006 (*Sneathia amnii*), OTU0007 (*Campylobacter ureolyticus*), and OTU0010 (*Bifidobacterium dentium*), while the OTUs that mainly belonged to the rectal swab samples were OTU0004 (*Escherichia coli*) and OTU0005 (*C. hominis*). The OTUs that were significantly elevated were shown by the LDA score, and OTUs with higher abundances had more LDA scores. Most OTUs that exhibited significant differences by LEfSe analysis are shown by the color blocks by different ethnic groups, sampling sites were rectal swab samples, and 80% of these OTUs exhibited differences among the six ethnic groups.

The three sampling sites and six ethnic groups selected in this study were very useful for studying the relationship and difference between the genital and digestive tracts among different ethnic groups living in the same city. Thus, PCA was performed using OTU data (**Figure 3B**) to confirm the microbial community differences in swabs from three different sampling sites from six ethnic groups. Most rectal swabs were clearly gathered together independently in PC1 (12.87%), PC2 (7.48%), and PC3 (5.50%). Vaginal and cervical swabs displayed a coincidence trend, and they were widely distributed in consideration of PC1 and PC2.

The difference between each pair of samples calculated by AMOVA was expressed by a distance matrix, and asterisks were used to indicate whether the difference was statistically significant (**Figure 3C**). A significant difference was primarily observed between rectal swabs and genital tract samples, while genital tract samples from the Yi and Lisu ethnic groups displayed less difference. Meanwhile, cervical swabs from the Naxi and Zang ethnic were distinct from vaginal swabs.

### 3.3 Identification of Microbiota Composition Markers Correlated With HPV Infection

**Figure 4:**
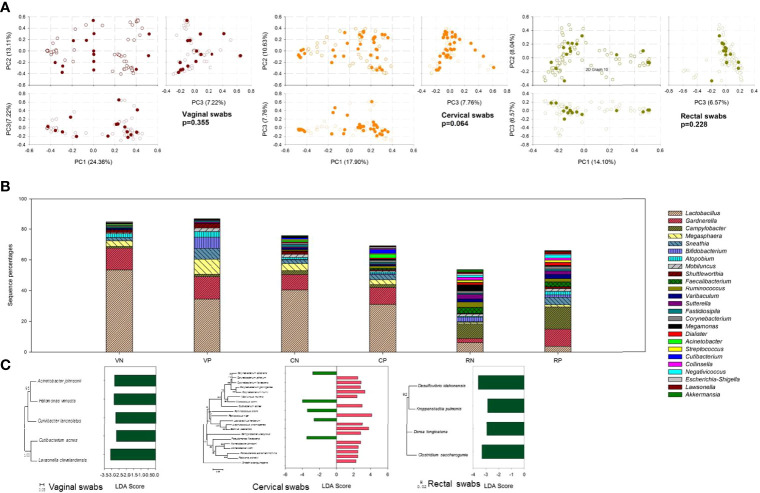
The effect of HPV infection on microbial community composition. **(A)** PCA plot based on the relative taxon abundance in vaginal, cervical, and rectal swab samples from HPV-negative (open circles) and HPV-positive (dark colored circles) subjects. **(B)** Microbial community composition at the genus level compared in vaginal, cervical, and rectal swab samples between HPV-negative and HPV-positive samples. VN, vaginal swabs from HPV-negative subjects; VP, vaginal swabs from HPV-positive subjects; CN, cervical swabs from HPV-negative subjects; CP, cervical swabs from HPV-positive subjects; RN, rectal swabs from HPV-negative subjects; RP, rectal swabs from HPV-positive subjects. **(C)** LDA value of LefSe analysis distribution histogram of the OTUs displayed significant differences between HPV-negative and HPV-positive samples.

To evaluate the variability of microbial communities between HPV-negative and HPV-positive groups, PCA was performed. Among the three swab types, there was no significant difference between HPV-negative and HPV-positive samples (p > 0.05) (**Figure 4A**).

Although statistical analysis yielded the above results, there were still some differences between HPV-negative and HPV-positive samples. At the genus level, as shown in **Figure 4B**, *Lactobacillus* was the most important biomarker in vaginal and cervical swabs, displaying much higher percentages in HPV-negative samples. More *Sneathia* and *Bifidobacterium* were detected in HPV-positive vaginal samples, but there was no significant difference in cervical samples. In rectal swabs, *Gardnerella*, *Campylobacter*, and *Sneathia* seemed to be more frequently detected in HPV-positive samples.

To identify species with significant differences between groups, LDA with LEfSe was used to identify the tagged species, namely, biomarkers. In **Figure 4C**, the average species abundance between HPV-negative and HPV-positive groups with significant p values (p < 0.05) is shown. In vaginal swabs, five biomarkers with p < 0.05 that showed similarity to *Acinetobacter johnsonii*, *Halomonas venusta*, *Curvibacter lanceolatus*, *Cutibacterium acnes*, and *Lawsonella clevelandensis* were found, all of which were significantly elevated in HPV-negative samples. In cervical swab samples, additional biomarkers were observed. The sequence percentages of *Corynebacterium accolens*, *Micrococcus cohnii*, *Ruminococcus bromii*, *L. herbarum*, and *Pseudomonas flavescens* were significantly higher in HPV-negative samples, while *C. jeikeium*, *C. flavescens*, *C. gottingense*, *C. ihumii*, *Mobiluncus mulieris*, *Cutibacterium acnes*, *Peptococcus niger*, *Staphylococcus chromogenes*, *Bacillus velezensis*, *Campylobacter ureolyticus*, *Acinetobacter johnsonii*, *A. lwoffii*, *Parasutterella excrementihominis*, *Ralstonia pickettii*, and *Sneathia sanguinegens* were more abundant in HPV-positive samples. The gut microbial composition was less affected by HPV infection, and only four biomarkers were responsible for HPV infection. *Desulfovibrio idahonensis*, *Kroppenstedtia pulmonis*, *Dorea longicatena*, and *Clostridium saccharogumia* were more abundant in HPV-negative samples.

### 3.4 Cytokine-Related Analysis

**Figure 5:**
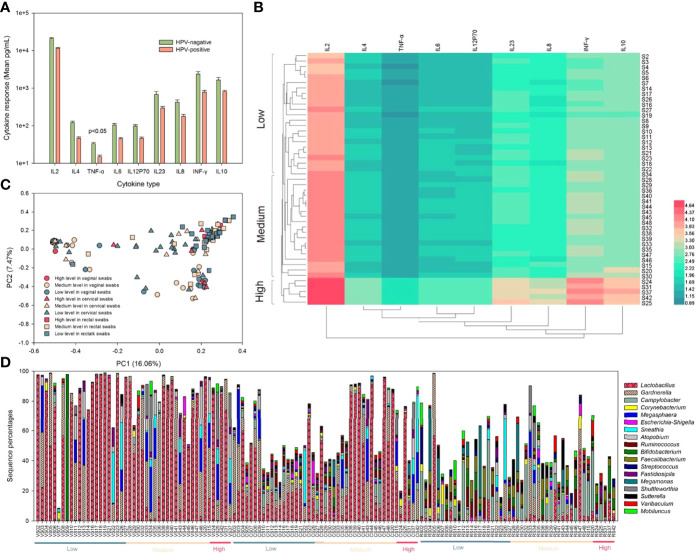
Cytokine determination, grouping, and effects on microbial community composition. **(A)** Cytokine determination with a logarithm of 10 in HPV-negative (light green) and HPV-positive samples (light pink). **(B)** All samples were clustered to low, medium, and high levels to determine correlation coefficients between individual cytokines according to cytokine levels (logarithm of 10) by average linkage cluster. **(C)** PCA plot based on the relative taxon abundance in vaginal, cervical, and rectal swab samples with low, medium, and high levels of cytokine clusters. **(D)** Relative microbial abundance determined using 16S rRNA gene sequencing clustered by low, medium, and high levels of cytokines in vaginal, cervical, and rectal swab samples.

In this study, nine cytokines were evaluated from a total of 47 volunteers’ serum; only one (TNF-α) was significantly (p < 0.05) downregulated in women with HPV, and all of those were lower in HPV-positive samples (**Figure 5A**). All samples were clustered to low, medium, and high levels to determine the correlation coefficients between individual cytokines according to cytokine levels (logarithm of 10) by average linkage cluster. All four samples with high cytokine contents were HPV negative (**Figure 5B**). PCA was used to confirm the microbial community differences among different cytokine levels (**Figure 5C**). Low- and medium-level cytokine samples were more dispersed than high-level samples. When analyzing the community composition of each sample, although there were individual differences, it was apparent that in the high-cytokine group, the content of *Lactobacillus* was low, while the content of *Gardnerella* was high (**Figure 5D**).

### 3.5 Relative and Absolute Quantities of Total Bacteria and Lactobacillus

**Figure 6:**
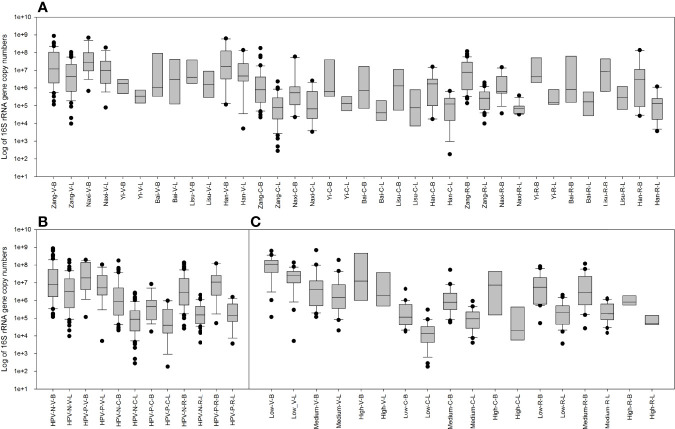
Copy numbers of the total bacterial and Lactobacillus 16S rRNA genes measured by quantitative real-time PCR. **(A)** Comparison between 16S rRNA gene copy numbers in the total bacteria and Lactobacillus among six ethnic groups in vaginal, cervical, and rectal swab samples. **(B)** Comparison between 16S rRNA gene copy numbers in the total bacteria and Lactobacillus among HPV-negative and HPV-positive samples in vaginal, cervical, and rectal swabs. **(C)** Comparison between 16S rRNA gene copy numbers in the total bacteria and *Lactobacillus* among low-, medium- and high-level cytokine clusters in vaginal, cervical, and rectal swabs.

The bacterial- and *Lactobacillus*-specific 16S rRNA genes were measured by quantitative real-time PCR (**Figure 6**). As expected, the amount of 16S rRNA gene in total bacteria was higher than that in *Lactobacillus*, which was lower in rectal swabs than in genital samples. The samples of each ethnic group were analyzed in detail and are shown in **Figure 6A**. In vaginal swabs, Naxi and Han women exhibited relatively high bacteria and *Lactobacillus*, while Yi women displayed fewer vaginal microorganisms. In most samples, the number of vaginal microorganisms was higher than that of the cervix, but the opposite trend was observed in the Yi samples.

HPV infection also affected the number of microorganisms (**Figure 6B**). In vaginal samples, HPV positivity increases the number of microorganisms and *Lactobacillus*. No significant differences were observed in the cervix or rectal samples.

When classified according to the level of cytokines (**Figure 6C**), low-risk cytokines exhibited a relatively concentrated and high number of microorganisms and *Lactobacillus* in the vagina. In the cervix, high-risk cytokine samples had a higher number of microorganisms and *Lactobacillus*.

## 4 Discussion

Biological susceptibility to HPV acquisition and immune competence for clearance of an HPV infection can be affected by vaginal microbial community composition, which disrupts the balance of the vaginal microbiota (McKee et al., 2019; Castanheira et al., 2021). Even healthy women were likely to suffer from HPV infection and vaginal microbial imbalance before they experienced symptoms. The current study systematically evaluated the genital tract health, the composition and ecology of the microbial ecosystem in the genital tract of fertile women, and the association between vaginal microbiota and HPV infection before symptoms appeared.

A high prevalence of HPV infection was observed in this study, which is a worrying phenomenon. The most common type worldwide in women with normal cytology in a large meta-analysis was HPV-16 (Bruni et al., 2010), which was also the case in this study. In the present study, infection with multiple HPV subtypes was not common, similar to other studies (Watson-Jones et al., 2013; Zhou et al., 2020). In addition, infection outside the cervix does not seem to be necessarily related to cervical infection. However, according to unpublished incomplete statistics by Obstetrics and Gynecology Department of Diqing Tibetan Autonomous Prefectural People’s Hospital, the incidence rate of CC is not high in the local area. This may be due to the following reasons. First, although it is said that Shangri-La is the hometown of longevity, this area is indeed an economically underdeveloped area. Many women lack relevant health knowledge, resulting in failure to go to the hospital for detection and diagnosis, even if they have CC. Therefore, HPV-positive people in this study will be further followed up to determine which of the above reasons is more likely. Second, lifestyles such as fewer sexual partners, older age of sexual life beginning, and lower number of births might also be related to HPV infection and resistance to the incidence of CC (Laniewski et al., 2020; Mei et al., 2022). Third, excellent local environmental advantages have created healthy reproductive system microbes, which are good for resisting the damage of viruses to human cells, causing a lower incidence rate of CC with a lower infection rate. However, it is speculated that the specific vaginal and cervical microecology might affect HPV infection in the cervical epithelium and the outcome after infection in a specific manner (Liu et al., 2020). Therefore, it is necessary to deeply study local women’s reproductive tract microorganisms.

Due to the physiological structure of the cervix and vagina, imbalance in the vaginal microecosystem directly increases the chance of HPV infection and accelerates the process of cervical precancerous lesions (Kovachev, 2020). Vaginal health is more commonly associated with low microbial diversity (Laniewski et al., 2020; Liu et al., 2020; Nunn et al., 2020), and there are some differences among the population. The reasons for these observed differences among ethnic groups are unknown, but it is tempting to speculate that the species composition of vaginal communities could be governed by genetically determined differences between hosts (Zhou et al., 2007; Zhou et al., 2019). These might include differences in innate and adaptive immune systems, the composition and quantity of vaginal secretions, and ligands on epithelial cell surfaces, among others. Previous studies have also shown that human habits and practices, including personal hygiene, methods of birth control, and sexual behaviors, exert strong influences (Zhou et al., 2007; Torcia, 2019). In Shangri-La, although the living environment is similar, people of different ethnic groups still have their own unique lifestyles, such as diet and hygiene habits.

In the female genital tract, the detection of Archaea was somewhat unexpected, but the following conclusions could still be drawn. First, the cervical samples had a high detection rate of Archaea, which might be related to the fact that the Archaea in this study was strictly anaerobic. Compared with the vagina, the oxygen content of the cervix is obviously lower and more suitable for the survival of methanogens (Yang et al., 2017). Second, although the detection rate of Archaea in rectal samples was higher, it is noteworthy that the distribution was not completely consistent with that in genital tract. However, only one subject had the same Archaea sequence in all three sites. Therefore, we might draw the conclusion that the Archaea in the cervix and vagina were likely to come from the gut. However, due to the difference in living conditions, the Archaea that can survive in these three parts at the same time might not be similar. The migration of Archaea in different parts of the human body and its relationship with HPV infection and clearance deserve further study.

Recent studies indicate a possible relationship between the gut and female tract microbiota, associating specific intestinal bacterial patterns with genital female diseases (Norman et al., 2007; Lindheim et al., 2017). Bacterial strains resident in the gut and vagina cross talk, which leads to local and systemic immune regulation (Quaranta et al., 2019). In this regard, the common microbial composition was observed between rectal and vaginal swabs in this study. In a previous study, we also found that the feces and amniotic fluid of newborns had the same bacterial composition with gut microbes of mothers (Liu et al., 2019). Indeed, understanding the connection between intestinal and vaginal microbiota might represent a goal for new treatments of female genital tract disorders.

The genital microbial composition could be involved in cervical oncogenesis and HPV clearance, which exist in a state of dynamic equilibrium, and homeostatic mechanisms exist to provide resilience. The abundances of *Gardnerella* and *Lactobacillus* were negatively correlated, especially in Han and Bai (lower percentages of *Gardnerella* and more abundant *Lactobacillus*). However, when *Gardnerella* is increased, it often indicates an imbalance in the microbial community in the reproductive system (Sierra et al., 2018).

Certain members of bacteria in the genital tract are believed to be beneficial for women against HPV infection. The primary defense mechanisms of the lower genital mucosa are antimicrobial peptides, a pH of less than 4.5, and a microbial community dominated by lactic acid producers. *Lactobacillus* species provide a key ecosystem service by producing lactic acid (Boskey et al., 1999; O'Hanlon et al., 2013), which is thought to restrict pathogenic organisms from colonizing the vagina (O'Hanlon et al., 2011). The abundances of lactobacilli and the overall composition of vaginal microbiota differ markedly between women (Zhou et al., 2007; Zhou et al., 2010; Ravel et al., 2011). In this study, although living in the same area, there were differences in living habits among several ethnic groups, even genetic information, especially in settlements divided by ethnic group. The lowest *Lactobacillus* sequence percentages and copy numbers in the vagina seemed to be related to the high number of births caused by their low education level, which made the vaginal microbial environment unhealthy. Interestingly, the opposite trend was observed in the Bai ethnic group. *Lactobacillus* was correlated with HPV infection, and Mitra et al. (Mitra et al., 2015) evaluated a group of 169 women referred for colposcopy and found increased bacterial diversity coupled with diminished lactobacilli that was associated with the severity of the cytological lesion. In this study, the sequence percentages of *Lactobacillus* in both the vagina and cervix were significantly decreased (**Figure 4B**); however, the copy number in vaginal samples was increased in HPV-positive samples and decreased in cervical samples. In the female genital tract environment, in addition to the microbial composition, the number of microorganisms is also closely related to health. After all, unlike the intestinal environment, which relies on a large number of microorganisms to help human digestion, once the delicate balance between the number and composition of microorganisms in the genital tract environment is broken, it can easily be invaded by various pathogenic microorganisms. Although *L. iners* was associated with a higher risk of being infected in a previous study (Norenhag et al., 2020), as the OTU with the highest abundance in this study, its abundance was not affected by HPV infection.

The abundance of some species was considered to be affected by HPV infection. Although the microbial community structures of the vagina and cervix were very similar, it was obvious that species that seemed to be related to HPV infection were not the same in the two environments. In the vagina, only a few species showed a positive correlation in HPV-uninfected populations. The relationship between microorganisms and HPV infection was more complex in the cervical environment because there were many species that exhibited significant differences. Moreover, these influential species appeared more frequently in HPV-positive samples. HPV infection seemed to increase the microbial abundance in the cervix. *Clostridium saccharogumia* and *Desulfovibrio idahonensis* have always been considered to be related to digestion in the intestine in their ability to catabolize mucosal carbohydrates and have a tendency to appear in HPV-negative populations (Hou et al., 2021).

Of note, the cytokines detected exhibited the opposite trend as in previous studies, and HPV infection led to decreased cytokine content (Kriek et al., 2016; Ondondo et al., 2020). However, cluster analysis revealed that there were still four samples with very high cytokine content (HPV negative), corresponding to the composition of the unhealthy microbial community. The reason for this phenomenon may be that our samples were not patients but virus carriers or patients with early infection, and they did not exhibit symptoms caused by viral infection. In addition, in this study, less than half of the subjects provided blood samples, and the small number also made the results in this study possibly have some deviation. Cytokines are associated not only with viral infection but also with microbial composition in the vagina and cervix and other health factors. However, this observation also suggests that HPV infection is mostly recessive and asymptomatic. Given the potential health hazards it brings to humans, regular detection and vaccination are very important.

Insights into the potential influence of the microbiome on viral persistence, immune response, host mucosal environment, and cancer treatments for HPV-related cancers are just beginning to emerge. HPV infection is associated with the vaginal and cervical microbiota. Hence, overall, microbes, environments, immune regulatory actions, and gene expression all interact closely to govern the homeostasis of the vaginal environment (Li et al., 2020). Our research confirms the following views: 1) There is indeed a difference in the microbial composition of the genital system of women from different ethnic groups, which might be more related to their lifestyle and subsequent living conditions. 2) *Lactobacillus* and *Gardnerella* were the primary bacteria present in the vagina. The balance between the two genera dictates the balance between the healthy and unhealthy states of the vagina. 3) The proportion of *Lactobacillus* in the cervix was much lower than that in the vagina, and the microbial composition was more complex in the cervix, so the complex virus–host–microbe interplays within the cervicovaginal microenvironment lead to complex metabolic potentialities (Ilhan et al., 2019; Li et al., 2022). 4) The proximity and connection between vaginal and gut microbiota allows for strong interactions between gut and vaginal bacteria. A refined equilibrium between gut microbiota, immunity, vaginal microbiota, and hormones would be more important than we thought in the physiological state of the female genital tract. 5) Asymptomatic HPV infection affected the microbial composition in both the vagina and cervix. In conclusion, microbial monitoring of vaginal and cervical environments is conducive to the identification of early HPV infection and other unhealthy factors. At the same time, modulating the microbial environment is another option for regulating vaginal and cervical health.

## Data Availability Statement

The datasets presented in this study can be found in online repositories. The name of the repository and accession number can be found below: The National Omics Data Encyclopedia; OEP002667.

## Ethics Statement

The studies involving human participants were reviewed and approved by the medical ethics committee of the First People’s Hospital of Yunnan Province. The patients/participants provided their written informed consent to participate in this study. Written informed consent was obtained from the individual(s) for the publication of any potentially identifiable images or data included in this article.

## Author Contributions

C-JL: data curation, writing—original draft. W-YX: data curation, writing—original draft. J-FF: cytokine and HPV determination. Y-HD: DNA extraction and PCR amplification. K-FY: sample preparation. M-PH: clinical samples collection. Y-SW: clinical sample collection. XL: sample preparation. Z-MZ: sequencing data analysis. TY: sample preparation. TZ: statistical analysis. C-YH: quantitative PCR. S-MZ: quantitative PCR. EY: statistical analysis. X-MW: funding acquisition, writing—review and editing, project administration. X-RL: funding acquisition, writing—review and editing, project administration. All authors contributed to the article and approved the submitted version.

## Funding

This study was funded by the Yunnan Province Innovation Team of Intestinal Microecology-Related Disease Research and Technological Transformation (China) (202005AE160010), Fundamental Application Research Foundation of Yunnan Province (2019FE001-174 and 2019FE001-298), and Major Science and Technology Projects (Biomedicine) in Yunnan Province (2019ZF004-1-5).

## Conflict of Interest

The authors declare that the research was conducted in the absence of any commercial or financial relationships that could be construed as a potential conflict of interest.

## Publisher’s Note

All claims expressed in this article are solely those of the authors and do not necessarily represent those of their affiliated organizations, or those of the publisher, the editors and the reviewers. Any product that may be evaluated in this article, or claim that may be made by its manufacturer, is not guaranteed or endorsed by the publisher.
