# Supplementary figures and images for: Genital Microbiota of Women From Six Ethnic Groups With and Without Human Papillomavirus Infection in Shangri-La, China
Source: Front Cell Infect Microbiol. 2022 Jul 8;12:935068. doi: 10.3389/fcimb.2022.935068 (PMC9304955; doi:10.3389/fcimb.2022.935068)

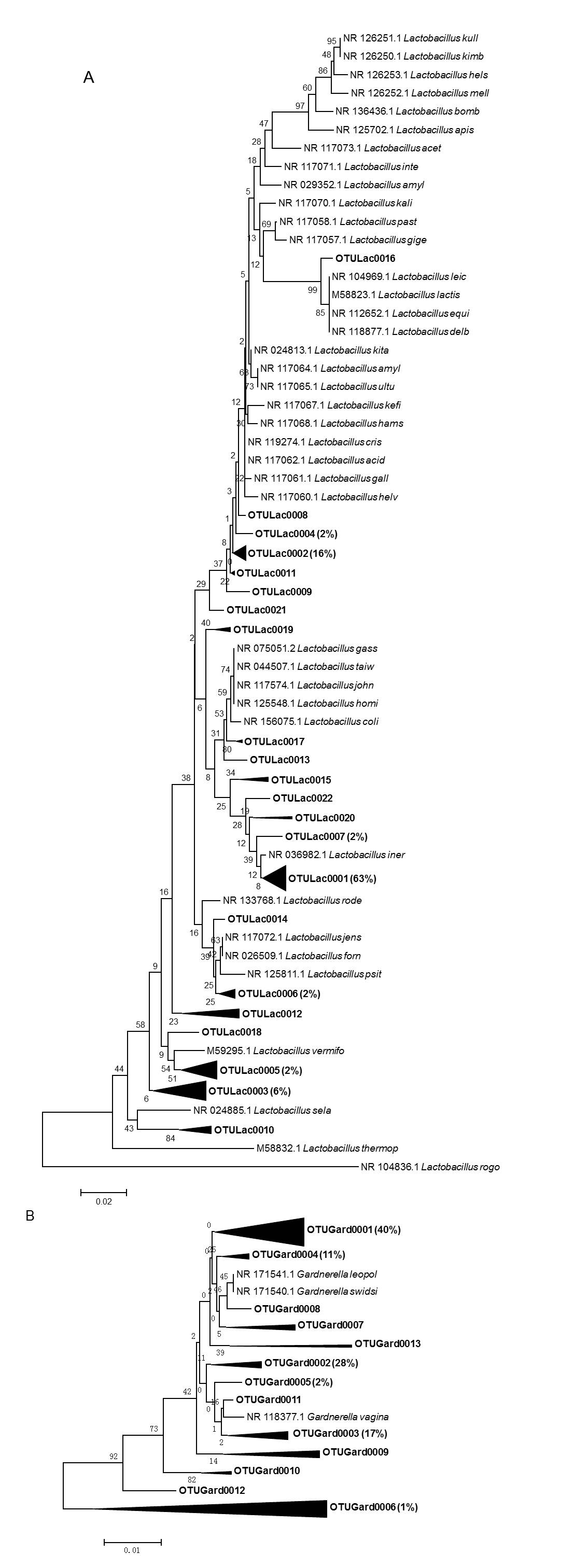

Supplement: Supplementary file 1 [file Image_1.jpeg]

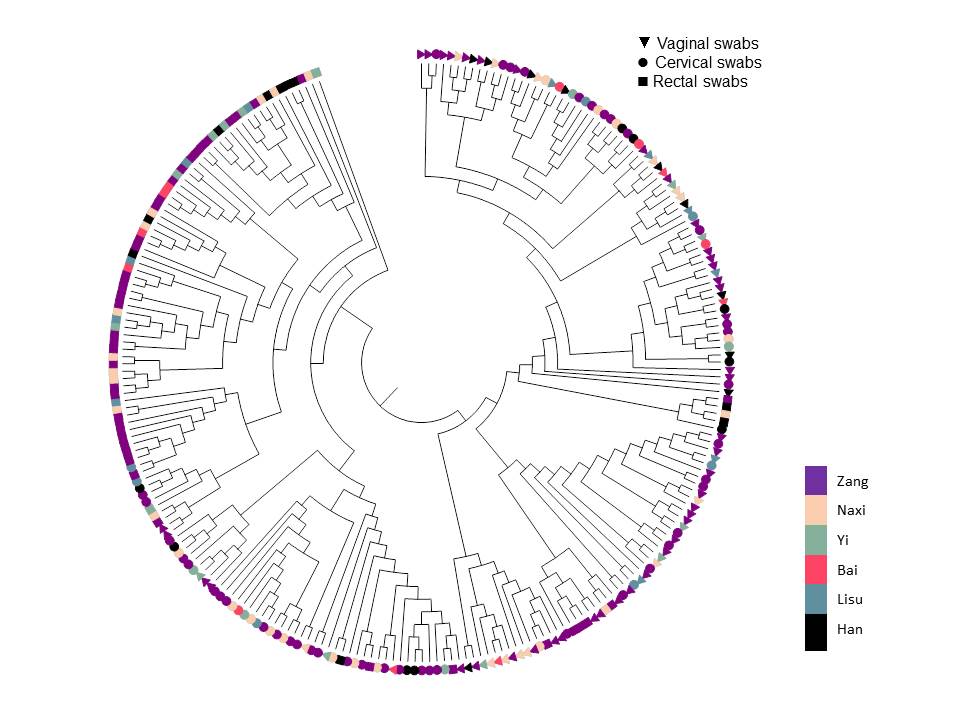

Supplement: Supplementary file 2 [file Image_2.jpeg]
